# Effects of Modulation Techniques (Manchester Code, NRZ or RZ) on the Operation of Hybrid WDM/TDM Passive Optical Networks

**DOI:** 10.1155/2014/984157

**Published:** 2014-10-30

**Authors:** Kumbirayi Nyachionjeka, Wellington Makondo

**Affiliations:** ^1^Department of Electronic Engineering, Harare Institute of Technology, Harare, Zimbabwe; ^2^Department of Information Technology, Harare Institute of Technology, Harare, Zimbabwe

## Abstract

In this paper, the performance and feasibility of a hybrid wavelength division multiplexing/time division multiplexing passive optical network (WDM/TDM PON) system with 128 optical network units (ONUs) is analysed. In this system, triple play services (video, voice and data) are successfully communicated through a distance of up to 28 km. Moreover, we analysed and compared the performance of various modulation formats for different distances in the proposed hybrid WDM/TDM PON. NRZ rectangular emerged as the most appropriate modulation format for triple play transmission in the proposed hybrid PON.

## 1. Introduction

Currently, telecoms operators are adapting their broadband access networks so as to enable these networks to support high bandwidth demanding services such as high-definition TV, interactive gaming and video conferencing. The exponential spread of internet into the remotest of areas in the world has led to hunger for larger data capacity. Recent increase in the demand for internet has brought focus on high bandwidth last mile access networks as these are the final hurdle to the consumer. Research into access networks has largely focused on ways to improve how customers can receive high definition television, videoconferencing and many other real-time services. As access networks and metro networks continue to converge, and research has begun to focus on dense and cost-effective optical access networks.

**Figure 1:**
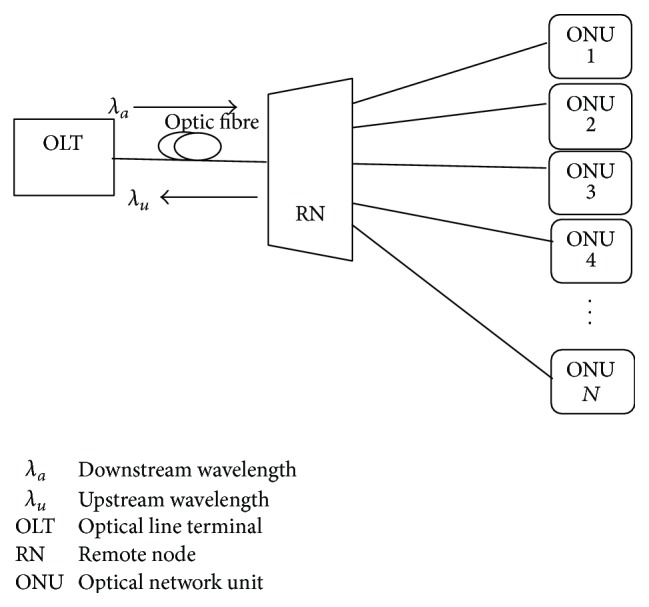
Passive optical network.

The advent of WDM and TDM systems, as a solution to increase the density of customers, is being realised, and the coming in of these systems has led to reduced cost as there is sharing of equipment in the optical distribution network and the optical line terminal (ONU). To further reduce the overall cost, the optic fibre network is continuously being made passive., In TDM passive optical networks, the TDM scheme is used to distribute the overall capacity among many users and because of this, each user's average bandwidth becomes limited to less than 100 Mb/s for GPONs and EPONs. In comparison, wavelength division multiplexing (WDM) PON is a strong alternative for future applications for access services requiring broadband. WDM PON provides dedicated transmission paths between each ONU and the OLT located at the CO, thus ensuring very fast connection for each subscriber with user localized bandwidth up to 10 Gb/s. A WDM PON has a maximum number of wavelengths that can be used, thus reducing the number of users that can be serviced, normally up to 64 users. To minimise these shortcomings of both the WDM PONs and TDM PONs. It is understood that the combination of these two PONs results in a hybrid network that is able to increase the number of users that an OLT can support with adequate bandwidth to every user.  Figure 1 [1] shows a PON set up with N users connected to the OLT.

Hybrid pons together with various modulation techniques can lead to improved systems in terms of performance and distance reached by the access networks. Hence, modulation techniques can thus enhance the performance of the hybrid system. Passive optical networks have shown that various reasons, chief among them low cost, high bandwidth support, simple operation, and maintenance, make them the mostly likely networks that are used for next generation access PONs.

## 2. Hybrid Passive Optical Networks (Hybrid PONs)

The explosion in internet traffic and ever expanding bandwidth requiring applications has led to the need for optic fibre networks to be deployed extensively in access networks area. The growth in these areas has become exponential and rapid that research is focussing on this area to provide the necessary degree of quality service to the consumer. In using purely TDM PON systems, speed can be the advantage obtained, but there is a limitation as to the number of users reached and also there is a limitation as to the amount of traffic a given user can access; this is because TDM uses one wavelength for downstream and another wavelength for the upstream communication limiting the optimum bandwidth for a given user, and thus the bandwidth for that particular single fibre is wasted, while on the other hand specific wavelength assignment for a given customer can be used in WDM PON which is an ideal and clear way of increasing capacity.

WDM is inefficient in its available spectrum utilisation because it lacks sharing of wavelengths. To deal with this inefficiency that arises from the purely WDM-PON and purely TDM-PON, further research and analysis into alternative access networks have to be done. This can be realised by combining the TDM PON layer with the WDM PON technology, thus increasing the capacity of the TDM PON. This increased capacity is due to the WDM PON, while the TDM layer allows the efficient spread of capacity to a larger number of customers. Therefore, the development of Hybrid WDM/TDM PON systems concept is imperative; hence, there is a need to enhance designs of a hybrid nature. Hybrid PONs architectures provide cost-effective, long reach, and large bandwidth to the customer. Therefore, research into different hybrid PON architectures used in conjunction with different modulation techniques and other performance enhancing solutions should also be investigated to further realise the best performance out of the hybrid PON.

### 2.1. Hybrid PONs with Predetermined Flexibility and Enhanced Performance

Increasing the bandwidth of access networks, serving a large number of users at minimum cost and fewer equipment, and covering the largest possible distance have become the most important goals in the last mile of fibre networks. This has been necessitated by the increase in the demand for triple play services like video, voice, and data. Now the advent of even improved quality of these services like 3D video, HD television, online gaming, an avalanche of social platforms, soaring online shopping, and advertising has led to a demand for larger bandwidth and fast speeds [2]. All the pure PON networks have been found to be inadequate to fulfil the needs of a next generation access PON network [3].

In [4], it was proposed a variety of hybrid WDM/TDM PONs whose operation depended on the flexibility of a predetermined remote node (RN). In a completely flexible hybrid network, simultaneous routing of a wavelength to any TDM PON or second remote node is done, thus providing the possibility of getting access to every TDM PON by any wavelength, which offered capacity on demand and reduced traffic congestion at the same time. The flexible system comes with constraints, such as the equipment cost due to the introduction of the active WSS (wavelength selective switch), that will increase the total cost and security issues will also arise due to the broadcast and multicast nature of this system. They also proposed partially flexible hybrid WDM/TDM passive optical network which uses the strength of both the flexible and the static systems. The static system has fixed wavelengths for each TDM PON, but it has low component count and increased security and suffers from traffic congestion.

Various investigations are done to continuously improve the aspects and marketability of hybrid pons, and in recent times the survivability of these networks has come into focus as a way of reducing data and business losses [5]. Also hybrid PON networks with a combination excluding TDM PON are also under investigation for future access network usage. OCDMA (optical code division multiplexing access) is a well-studied area and a pure OCDMA is valued for its unique properties like fully asynchronous access capability, high security, and soft capacity-on-demand, and this combined with the properties of optic fibre and the qualities of WDM PON leads to competitive option for optical access networks [6]. A hybrid WDM/TDM GPON using radio over fibre technique was reported in [1] and a hybrid GPON using RoF with 2.5 Gbps, digital modulation (8DPSK), and 2.4 GHz was implemented and this gave good performance over 25 km of fibre length for 32–64 users with a good OSNR. The working of a hybrid WDM/TDM PON depended on the architecture of the network and so does its performance. In [7], it was reported how the performance of a hybrid PON can be improved by using a tunable wavelength converter based switching ring converter structure (TWCSS). It is demonstrated that 64 ONUs × 16 wavelength system worked properly with reduced loss of packets; the average packet delay was the shortest and increased access capacity was also realised.

A spectrally efficient design [8] was demonstrated with a centralized light source hybrid WDM/TDM-PON that supported 160 ONUs with a data rate of 625 Mbit/s downstream and 156 Mbit/s upstream for each customer, and it was shown that the spectral efficiency of 50 GHz channel spacing can be implemented over a distance of 20 km. A host of advantages were noticed such as good constellation diagrams, clear and wide eye diagrams, and low transmission power penalties on receiver sensitivity in both upstream and downstream transmissions. Study has also been done in using mathematical algorithms to aid in the design of the most effective hybrid PON in [9]. They proposed a location/allocation algorithm (LAPON) whose nature is three phases. This algorithm provided ways to physically arrange the equipment without naming the type of the equipment. It also provided insight into how the ONUs should be physically cascaded into PON network architecture and it also provides the probable location of the splitters or AWG.

Kim et al. [10] demonstrated a hybrid PON that uses colourless RSOA-based 32-channel loopback WDM PON that satisfies backward compatibility and this was able to support a reach of about 50 Km and 128 split ratios for a given wavelength and error-free upstream and downstream communication was achieved at injection power in excess of −17 dBm. A hybrid WDM/TDM EPON using a dynamic wavelength and bandwidth-allocation algorithm (DWBA) was discussed in [11]. In this system the ONUs are required to report their bandwidth requirement status with respect to their priority queues. An adaptive linear prediction method was investigated to compute the average arrival rate of variable bit rate flow during the next waiting interval for a given ONU. This enabled the procedure to assign bandwidth and wavelength effectively. It was shown that using this algorithm significantly improved the delay jitter performance, thus improving the quality of video streaming sent to the customer. Different modifications have been carried out to try and improve the performance of hybrid PONS, and the target was to increase the split ratio and achieve the maximum possible reach by these access networks. One such proposal was the designing of a WDM-PON/TDM-PON with self-homodyne system employing R-SOA as the transmitter in the colourless domain. Balancing the receiver eliminates the beating noise which is due to seed light reflected; also the variation in phase due to the coherent detection is done by the balanced receiver. Thus, it was seen that power loss budget was much improved due to the total suppression of the reflection noise and this impacted well on the overall performance of the hybrid PON [12].

Wavelength specified laser can be used. It offers an advantage by generating multiple wavelengths since there is no probable gain that will be taken advantage of by the different wavelengths supporting ONUs, it also helps in the probable multiplexing of flow from every ONU leading to improved performance of the system, enabling the colourless characteristic of the ONUs greatly aids with added simplicity the management of the inventory, minimises costs for spares and enhances the automated provisioning of wavelength [13].

In [14] signals were communicated over 26 km. Each signal was 1.25 Gb/s and this was done over a single mode fibre promoting dual opposing traffic to the user. This analysis was done on a hybrid WDM/TDM PON access network which was built using low cost equipment. A polymer Bragg reflector fitted to a TECL with the modes spaced at 0.8 nm has 25 channels directly modulated at 2.5 Gb/s for use as sources with low cost in WDM-PON systems. It was reported that the tunable external laser with directly modulated transmission at 2.5 Gb/s over a single mode fibre of length 20 km was implemented with success [15]. GEPON design utilising a 1-to-8 optical splitter was used in [16]. The design used the elements of the passive optical network to connect the CO and different customers. The design was investigated for different lengths between the CO and the PON up to a distance of 15 km and the parameter under observation was the BER.

Wason and Kaler [17] came up with an algorithm for efficient assigning of wavelengths in light path dynamic provisioning. This system was based on the concept of the most frequently wavelength used, to effectively reduce the probability of blocking. The system was then compared with those systems that were already available and it was found that it gave more accurate results.

### 2.2. Methodology

**Figure 2:**
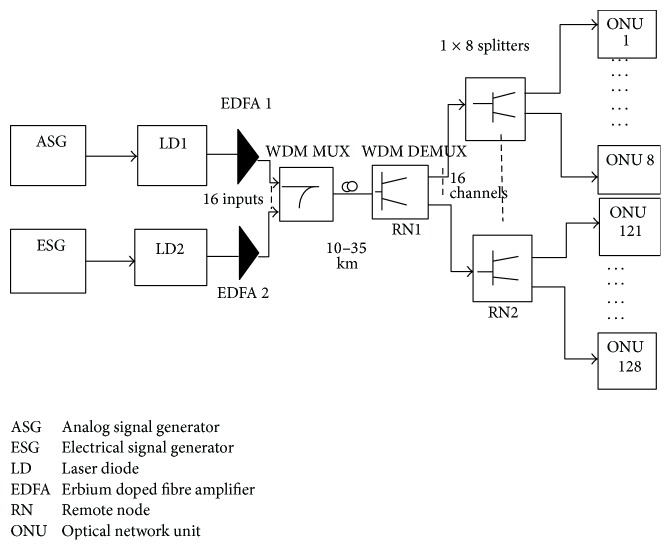
Block-diagram for hybrid optical network.

The simulation setup for the hybrid network is as shown in Figure 2. In the envisaged system, 128 customers can utilise video and voice/data for a given distance of 28 km in the absence of a repeater. OLT components are placed at the central system, while at the customers end ONT components are placed there. Branch architecture is employed for fibre distribution. 128 customers are connected to the OLT by splitting the fibre to 16 × 8 times. A quick glance at the literature survey suggests that video, data, and internet are also known as triple play. The CO unit is where video, data, and voice signals are realised. The downstream data components are obtained by a data link of 1.25 Gb/s bandwidth and the signals are generated by pseudo random data generator (PBRS) and an electrical signal generator. Voice is represented as voice over IP (VOIP), packet switched protocol. Voice and data are combined and transmitted using either Manchester code, RZ or NRZ modulation formats. Eight wavelengths with spacing of 0.8 nm are used within the range 1480–1500 nm and transmitted, each through a direct modulated laser and a booster amplifier. RF SCM system with eight channels is used to represent video components at 0.8 Gb/s bandwidth; these channels are within the frequency range 55.25 MHz–547.25 MHz and psk modulation is used in these channels for video transmission. Two analogue signal generators, electrical adder, direct modulated lasers and a preamplifier, are used to generate each of the video signals which are then transmitted within the 1550 nm–1560 nm range. A preamplifier is then used to strengthen the signals before transmission and in this case an EDFA is employed. An optical combiner is further used to combine the video and data/voice to come up with a signal that is launched into the channel. The signal has to go through two remote nodes before it gets to the ONU. The first remote node is equipped with a fiber trunk that varies in length between 10 km and 35 km, and the fiber is also split by using a 1-to-16 splitter. The 16 outputs travel to the second remote node over a fiber of 1 km. The RN2 uses 1-to-8 splitter and is used to supply 8 separate channels. The second RNs are at least 300 m from the users (ONUs), while the second remote nodes are 1 km from the first remote nodes. The first remote node is connected to the transmitter at the central office by a 10–35 km varying fiber trunk. A power splitter, delay blocks, and pulse train generator are found at the second RN. A delay of 10 ps is instituted in the second RN for every signal arriving there, while those arriving at the first node have zero delay, and so forth. At the ONU, these signals are further separated by a demultiplexer into a video signal and data/voice signal by way of a Fabry-Perot optical filter, whose centre wavelength varies depending on the service the user requires. Finally, an APD is used to detect the signals.

### 2.3. Results

**Figure 3:**
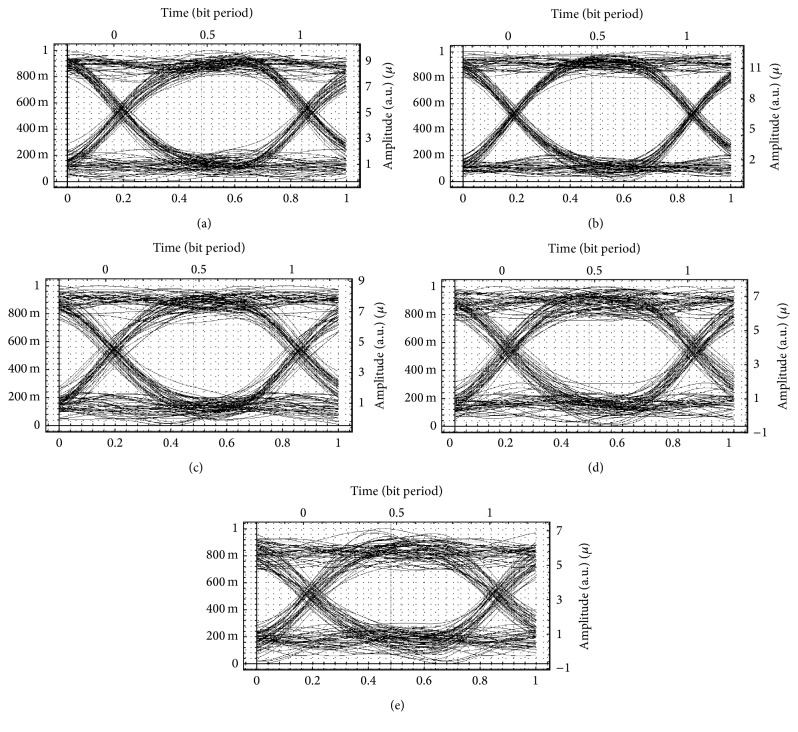
Eye openings for a hybrid network system with 128 customers using the nonreturn-to-zero format at (a) 19 km, (b) 22 km, (c) 25 km, (d) 28 km, and (e) 31 km distance, respectively.

Different modulation techniques were used to transmit video and voice/data over a fiber cable, so simulation is done for different distance and produces various waveforms. The BER and quality factor (*Q*) for different fiber distances are computed and eye opening graphs are seen. Figures 3(a)–3(e) show the eye openings for 128 customers simulated with nonreturn-to-zero for 19 km, 22 km, 25 km, and 28 km. Clearly from the eye openings, it is seen that communication distance is improved to 28 km, and going above this distance, noise increases drastically and transmission is inhibited. Hence, the services can be utilised for 28 km only.

**Figure 4:**
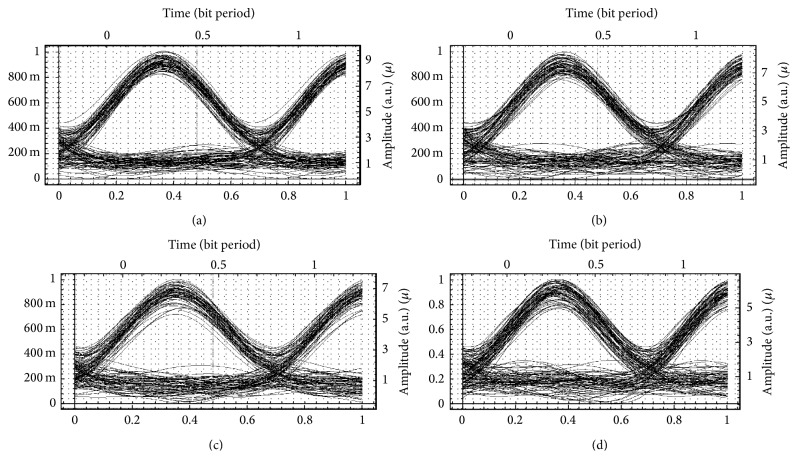
Eye openings for a hybrid network system with 128 customers using the return-to-zero format at (a) 19 km, (b) 22 km (c) 25 km, and (d) 28 km distance, respectively.

Figures 4(a)–4(d) show the eye openings for 128 customers simulated with return-to-zero for distances of 19 km, 22 km, 25 km, and 28 km. Clearly from the eye openings, it is seen that communication distance is improved to 25 km, and going above this distance, noise increases drastically and transmission is inhibited. Hence, the services can be utilised for 25 km only.

**Figure 5:**
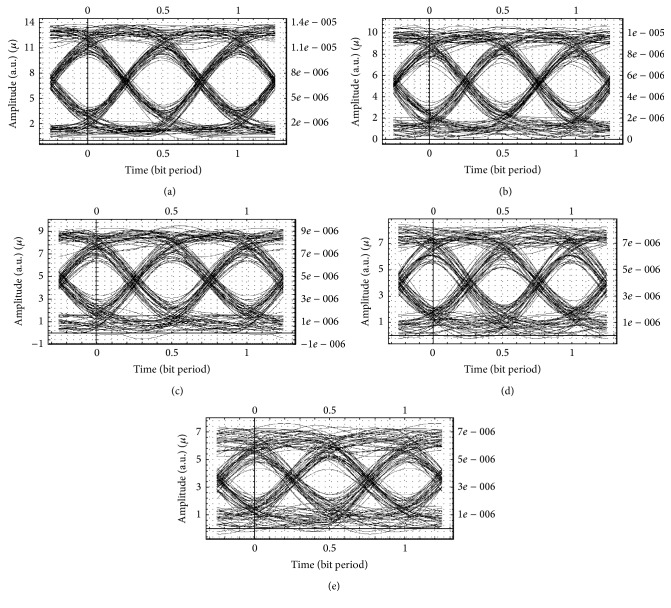
Eye openings for a hybrid network system with 128 customers using the Manchester code at (a) 10 km, (b) 16 km, (c) 19 km, (d) 22 km, and (e) 25 km distance.

Figures 5(a)–5(e) show the eye openings for 128 customers simulated with Manchester code for distances of 10 km, 16 km, 22 km, and 25 km. Clearly from the eye openings, it is seen that communication distance is improved to 16 km, and going above this distance, noise increases drastically and transmission is inhibited. Hence, the services can be utilised for 16 km only.

BER and quality factor computed results at various distances for the three modulation formats used in a hybrid PON system for 128 users and this is given in Tables 1, 2, and 3.

**Figure 6:**
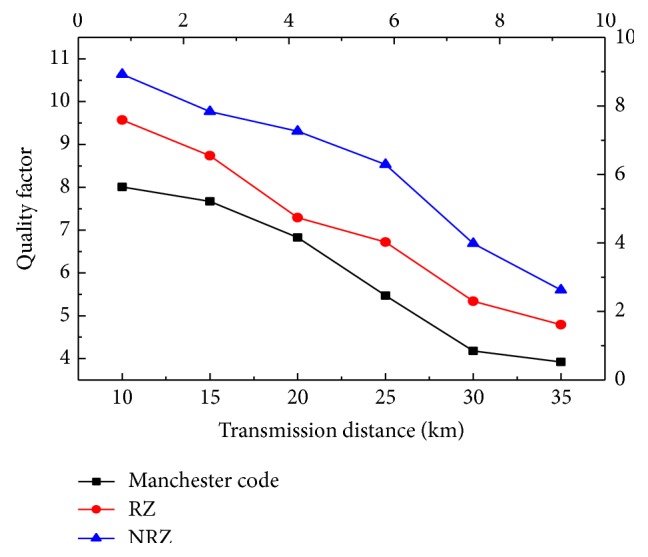
Distance in kilometres versus quality factor, *Q*.

**Figure 7:**
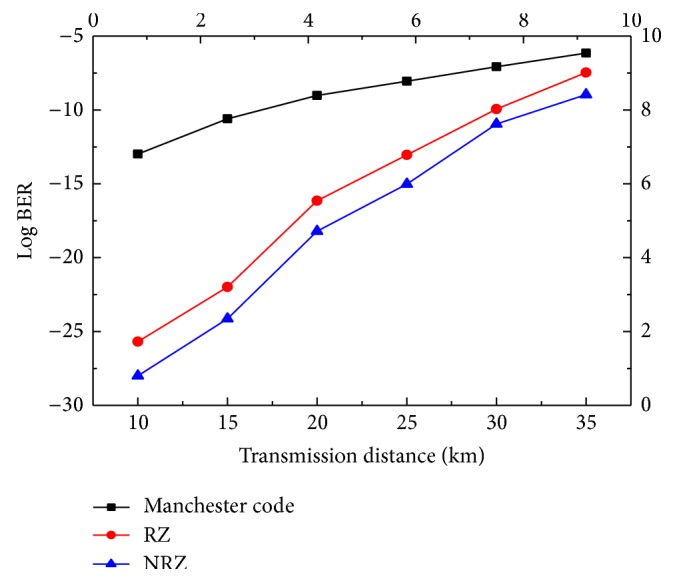
Distance in kilometres versus Log of BER.

The optic fiber length was varied between 10 km and 31 km.  Figure 6 shows the quality factor (*Q*) versus distance graph for hybrid PON system. It is seen that the minimum *Q* value for acceptable transmission to the user is reached at 19 km, 25 km, and 28 km for all three modulation formats for 128 users. Increasing the distance beyond 35 km reduces the *Q* value and hence the ability of the system to transmit the triple play services is reduced. Triple play services in hybrid PON can best be transmitted by using the NRZ modulation format.  Figure 7 shows the transmission distance in kilometres versus Log BER for the hybrid passive optical network for 128 users. A transmission distance range 10–35 km is used in this graph. It is observed that the least BER is obtained up to 19 km, 25 km and 28 km for Manchester code, RZ and NRZ respectively. If distance is increased beyond these the BER increases and the system fails to communicate the triple play services.

## 3. Conclusion

Hybrid PON based FTTH with 128 customers was simulated. The customers were able to receive triple play signals through a single optic fiber. A range of wavelengths between 1480 nm and 1500 nm is used to communicate voice/data signal at 1.25 Gb/s and a range of wavelengths between 1550 and 1560 nm is used for video signals with 0.8 Gb/s. Based on these results, it is evident that NRZ rectangular provided the longest transmission distance making it the most ideal for the transmission of the signal. The split ratio of passive networks can be greatly increased by hybrid optical networks as evidenced in this work.

Improvement may still be done to better understand hybrid networks such as (a) to improve the split ratio, (b) to increase the reach of the PON, (c) to enhance component performance, in particular power output of the source and the sensitivity of modulation, and (d) to analyse the system in terms of the wavelengths.

## Figures and Tables

**Table 1:** Calculated BER and *Q* values for NRZ.

Distance (km)	*Q* factor	BER
16	10.64	1.02*e* − 26
19	9.77	7.39*e* − 23
22	9.31	6.15*e* − 21
25	8.53	7.53*e* − 18
28	6.69	1.13*e* − 11
31	5.60	1.064*e* − 8

**Table 2:** Calculated BER and *Q* values for RZ.

Distance (km)	*Q* factor	BER
13	9.57	2.089*e* − 26
16	8.74	1.0551*e* − 22
19	7.29	7.075*e* − 21
22	6.72	5.597*e* − 15
25	5.34	1.1221*e* − 10
28	4.79	3.419*e* − 9

**Table 3:** Calculated BER and *Q* values for Manchester code.

Distance (km)	*Q* factor	BER
10	8.01	1.03*e* − 13
13	7.67	2.5*e* − 11
16	6.83	9.5*e* − 10
19	5.47	8.7*e* − 9
22	4.18	8.5*e* − 8
25	3.92	6.91*e* − 7
